# Spectroscopic Monitoring and Modeling Drug Dissolution for Undergraduate Chemistry Curriculum

**DOI:** 10.1021/acs.jchemed.2c00707

**Published:** 2024-03-12

**Authors:** Chengxuan Guo, Nicole Wendel, Ally Lee, Shonda Monette, Brian Morrison, Dominic Frisbie, Earlene Erbe, Renée S. Cole, Max Lei Geng

**Affiliations:** † Department of Chemistry, University of Iowa, Iowa City, Iowa 52242, United States; ‡ Chadwick International School, 45, Art center-daero 97 beon-gil, Yeonsu-gu, Incheon 22002 South Korea

**Keywords:** Upper-Division Undergraduates, Analytical Chemistry, Inquiry-Based/Discovery Learning, Drug/Pharmaceuticals, Kinetics, Laboratory Equipment/Apparatus, Quantitative Analysis, UV−vis Spectroscopy

## Abstract

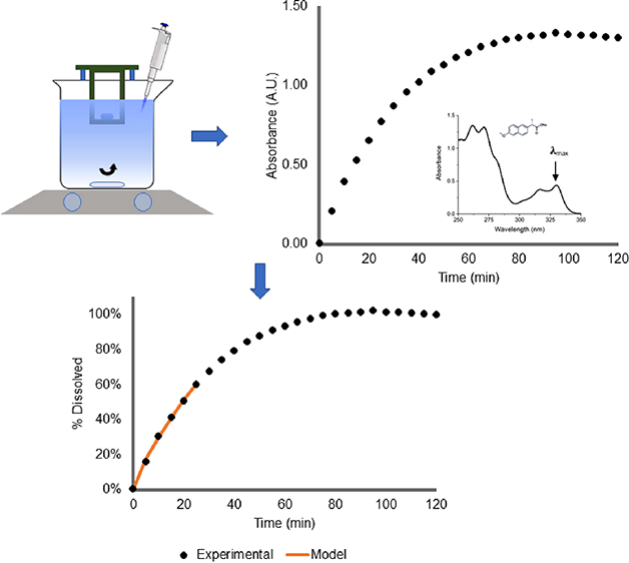

The pharmaceutical and medicine manufacturing industry has become the largest industrial sector for the employment of chemists, indicating a need for experiments with a pharmaceutical sciences context in the undergraduate chemistry curriculum. In the pharmaceutical industry, testing drug dissolution is a key analytical task for solid oral dosage forms that is performed in different phases of drug development to test the release behavior of new formulations, ensure consistency between manufacturing lots, and help predict the *in vivo* absorption of the drug substance after administration. However, there are a limited number of laboratory experiments in dissolution testing developed for the undergraduate chemistry curriculum. To help students obtain hands-on experience in dissolution testing, a protocol has been developed for an undergraduate chemistry laboratory course for students to build a dissolution apparatus, monitor dissolution processes, model the dissolution to extract kinetic parameters, and evaluate the consistency between dissolution curves with FDA regulated methods. Students successfully collected dissolution curves and completed the modeling analysis with nonlinear least-squares fitting. The designed dissolution protocol has been evaluated to have consistency and reproducibility to be implemented in the undergraduate chemistry laboratory curriculum.

Drug dissolution and release methods to achieve effective drug delivery have been extensively studied in pharmaceutical research.^1−5^ The monitoring of dissolution and release is essential to the evaluation of new drug formulations and guides the development processes.^6,7^ The dissolution of a drug from the solid oral dosage form is the beginning point for the subsequent diffusional mass transfer in human body fluid. It involves breaking down solid-state intermolecular forces of the tablet matrix upon contacting dissolution medium, followed with the active pharmaceutical ingredient released from the matrix and diffusing into the dissolution medium.^8,9^ In the pharmaceutical industry, dissolution testing is a central process for quality control and new formulation development of solid dosage drugs.^10−12^ Considering the fact that a significant fraction of chemistry graduates will be working in the pharmaceutical sector,^13,14^ knowledge and experimental experience of dissolution testing methods provide good preparation of undergraduate students majoring in chemistry and related fields. In the pharmaceutical industry, *in vitro* drug dissolution tests are typically performed with dissolution devices authorized by and following the standard protocol developed by the United States Pharmacopeia (USP).^15^ Chemistry education studies have been conducted for implementing pharmaceutical analysis in teaching laboratories, particularly in the identification^16−19^ and quantification of drug molecules^20−26^ and introduction to drug design.^27−30^ However, there are a limited number of laboratory experiments in dissolution testing developed for the undergraduate chemistry curriculum;^31,32^ USP dissolution apparatuses are expensive, and it is not feasible to provide one instrument for each student group in the teaching lab. We decided to design a cost-effective apparatus and develop a dissolution protocol for the undergraduate teaching laboratory. In summary, the goals of this experiment are for students to

- Build a dissolution apparatus and perform dissolution testing, generating dissolution curves with UV spectrophotometry data.
- Use nonlinear least-squares (NLLS) analysis to fit the dissolution profiles with a widely used dissolution model.
- Evaluate the consistency in dissolution profiles per FDA regulated criteria.
- Have an opportunity to experience protocols related to pharmaceutical research and development.

## Development of Dissolution Protocols

### Experimental Design

To identify an appropriate drug molecule for the dissolution experiment, we searched the pharmaceutical literature on the dissolution curves of medication and supplemental products that are generally used in daily lives, including nonsteroidal anti-inflammatory drug (NSAID) molecules acetaminophen, ibuprofen, and naproxen and vitamins. The selection of a molecule that students are familiar with was intended to facilitate a classroom discussion of using the dissolution measurements to predict and substitute for *in vivo* release monitoring. With the literature dissolution profiles on hand, we applied a number of criteria in selecting the final drug candidate: (1) The dissolution process takes between 60 and 90 min to reach 95% dissolved, a time that is long enough to allow for the collection of a sufficient number of data points on the dissolution curve to allow mathematical fitting to dissolution models and short enough to fit into a single lab period of 2 h and 50 min. (2) Once dissolved in a 1 L solution, the drug molecules would generate a molecular absorption signal that is within a range of good signal-to-noise ratio to teach students measurement principles. (3) The absorption can be measured in the visible region of the electromagnetic spectrum to allow the usage of disposable plastic cuvettes for the large undergraduate laboratories.

**Figure 1:**
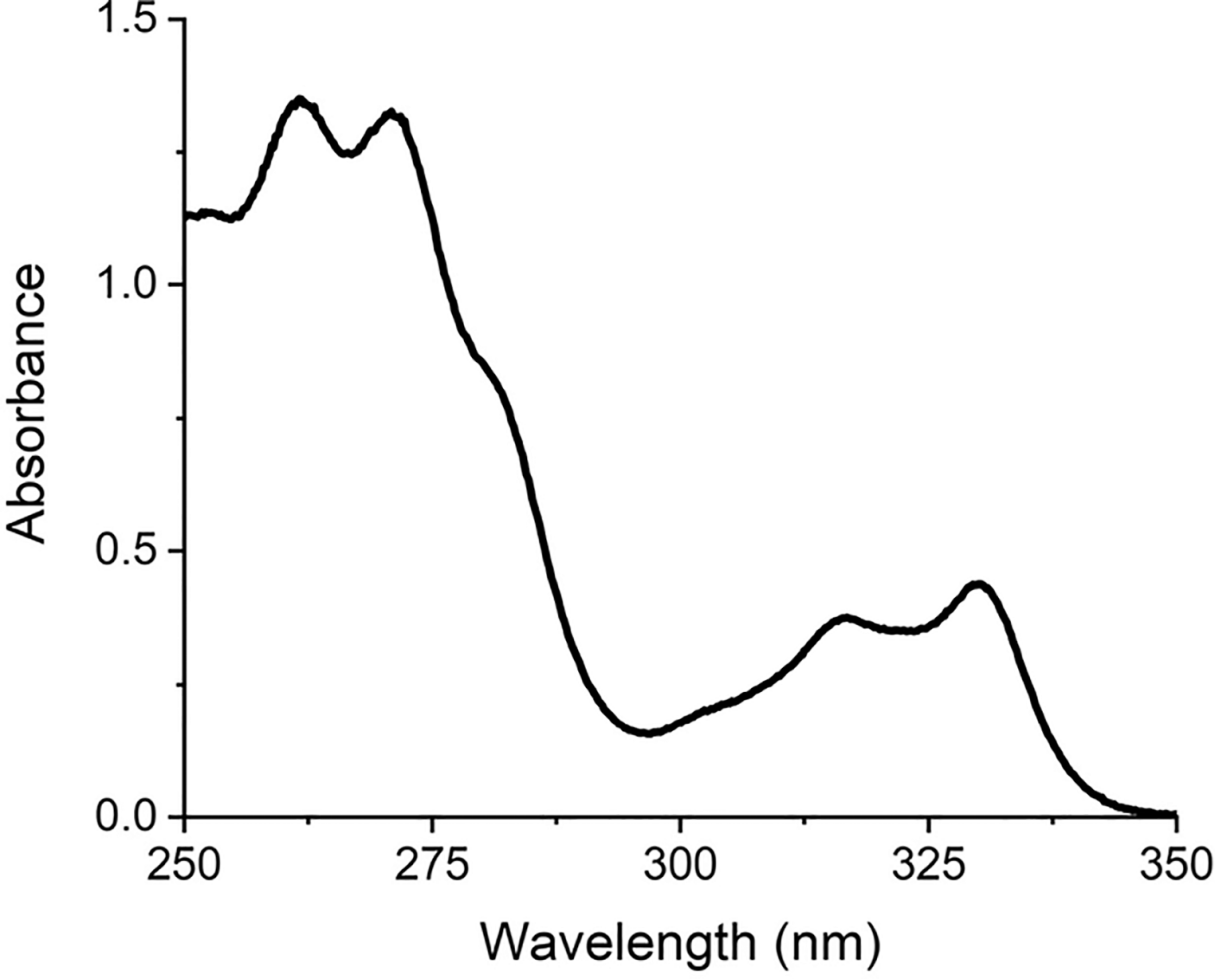
Absorption spectrum of naproxen sodium.

The drug selection proved to be challenging. Most over-the-counter drugs are designed to take effect quickly, and their dissolution typically completes within 20 min. We estimated that the fastest sampling and data acquisition in the experiment should be 5 min per data point; as each sampling involves the accurate removal of an aliquot of the dissolution solution, accurately replenishing the solution, and spectrophotometric measurements, 5 min intervals seem reasonable for undergraduate students enrolled in the course. This proved to be appropriate throughout the undergraduate experiments in several semesters, with students having just enough time to complete all of the experiment steps between data points. Additionally, we preferred the collection of up to ∼20 data points on the dissolution curve for good model fitting, especially when the kinetic models typically apply to only the first 60% of the dissolution process, resulting in 6–8 data points available for fitting (*vida infra*). To fit an experimental curve to a nonlinear model containing two parameters, fewer data points would not be proper. These constraints require the total dissolution time to be ∼100 min. We discovered that naproxen sodium, the active ingredient in Aleve, is the most appropriate molecule for the experiment. The UV–visible absorption spectrum of naproxen sodium (Figure 1) shows four major absorption peaks in the UV region. The common dosage of naproxen sodium is 200 mg per tablet. Once completely dissolved in 1 L of solution, the absorbance of the solution exceeds 2.0 at 272 nm, the wavelength of absorption maximum for assaying naproxen sodium reference standard per USP monograph,^16^ and cannot be measured with the spectrophotometer used in our undergraduate laboratory. Additionally, measurements of absorbance at 2.0 are associated with low signal-to-noise ratio (SNR) and are not preferred in the teaching of chemical measurements. Multiple sources for deviation from the Beer’s Law are discussed in lectures, and students understand that at these high levels of absorbance there is a possibility that the data would deviate from linearity predicted by Beer’s Law. We thus followed the dissolution at the 329 nm transition per USP dissolution protocol for naproxen sodium tablets^16^ and opted to use the opportunity to teach students how to be careful in conducting the experiment and not break the expensive quartz cuvettes.

**Figure 2:**
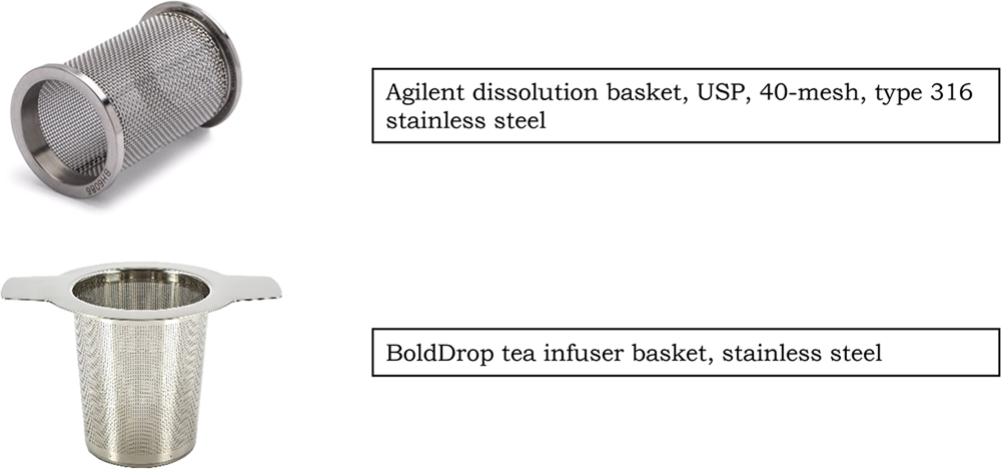
USP dissolution basket and the proposed alternative basket for the undergraduate laboratory session. (Model details and web sources are in Section I of the Supporting Information).

The dissolution basket for the USP protocols is defined by standard dimensions and mesh sizes stipulated by the USP. A typical dissolution basket shown in Figure 2 is made of stainless steel and has 40 mesh sieves with 381 μm openings. At ∼$150, these baskets are costly for large undergraduate lab courses. In searching for a dissolution basket for the experiment, a number of criteria were considered: (1) the pores of the basket need to be small enough to block large particulates from going through yet large enough not to impede the diffusion of the dissolved drug molecules into the bulk dissolution solution in order for the kinetic profile to be reliably measured, (2) the basket needs to be constructed with high structural standards such that the results between separate baskets are consistent, and (3) the baskets should be structurally rigid so they will not deform or change shape. In the dissolution process, the drug tablet breaks apart into small pieces and particulates, each containing undissolved drug molecules. If these particulates were allowed to go through the pores of the basket, they would contribute to the spectroscopic signal of the dissolution solution and give rise to erroneous kinetics. The small pore requirement in Criterion (1) thus ensures the reliable kinetic measurements.

After extensive searching, a tea infuser basket (BoldDrop Inc.) proved to be a good selection for our undergraduate laboratory as it satisfies all the criteria: it is constructed with stainless steel, has a rigid structure that does not deform in undergraduate laboratories, has a repeatable structure between baskets to facilitate comparison between student groups, possesses pore sizes that would not impede molecular diffusion while securely enclosing the tablet fragments, and is of low cost. Added features that proved to be beneficial are the two wings that facilitate the assembly of the entire dissolution device and a lid that completely covers the basket during the experiment to prevent solvent evaporation during long kinetic measurements.

Although the device of choice shares the structural properties with the USP designed dissolution baskets, clearly, the dimensions of the basket are not identical with those mandated by USP. This could affect the curve feature and rates of the measured dissolution. However, the objective of the experiment is to teach students the principles of drug dissolution measurements, and the basket of choice provides a robust and cost-effective solution to our instructional objective.

### Chemicals and Materials

Naproxen sodium tablets obtained from Albertsons, Inc. (Phoenix, AZ) were used as the sample to develop the dissolution procedure as the completion of the dissolution is typically around 1 h,^15^ which fits in the time length of one lab session. Naproxen sodium solid acquired from Sigma-Aldrich (St. Louis, MO) was used to prepare standard solutions for measuring the molar absorptivity through calibration and for monitoring potential instrumental drift during the long kinetic measurements. Millipore deionized water was used as the dissolution medium and the solvent for solution preparation.

### Dissolution Apparatus

**Figure 3:**
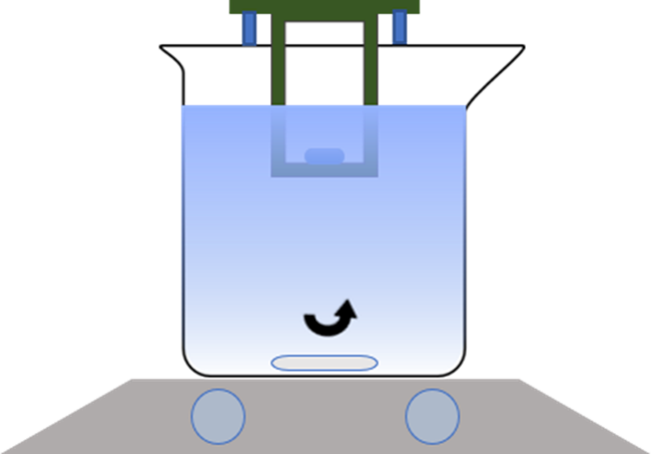
Designed dissolution apparatus. Components included: a dissolution vessel, dissolution basket, aluminum bar supporter, magnetic stir bar, and a stir plate. The two circles represent the stirring and heating knobs on the stir plate.

The dissolution apparatus was designed to be composed of a dissolution vessel, a dissolution basket, and an agitation system. A 1 L glass beaker was selected as the dissolution vessel to be filled with the dissolution medium. This volume is consistent with those used in the USP dissolution devices and protocols. A stainless-steel fine-filtering tea infuser was used as the dissolution basket. To assemble the apparatus, a magnetic stir bar was placed at the center of the bottom of the 1 L beaker. The vertical axis of the dissolution basket was aligned at the center of the beaker and supported by two aluminum bars, prepared from the Chemistry Machine Shop, to be placed on the rims of the beaker. The aluminum bars were 6 in. *L* × 3/4 in. *W* × 1/8 in. *H* in dimension and polished to remove all sharp edges after fabrication. The position of the basket was secured with masking tape. The schematic of the apparatus is shown in Figure 3.

### Dissolution Protocol

Spectroscopic monitoring of the dissolution of naproxen sodium tablets was performed by measuring the solution absorbance at sequential time points with a portable USB4000 UV/vis Spectrophotometer (Ocean Optics, Inc., Largo, FL). To develop the protocol of the dissolution measurements, experimental conditions were optimized as described in Section II of the Supporting Information. In the final procedure, Millipore water was used as the blank to calibrate the spectrophotometer. A large stirring bar was placed in the center of the bottom of the dissolution vessel. The dissolution apparatus was quantitatively filled with 900 g of water. The dissolution basket was placed in the center of the vessel, supported by the two aluminum bars. The entire device was covered with parafilm to prevent solvent evaporation during the ∼100 min kinetic measurements. An opening was left between the rim of the dissolution vessel and the release basket to allow periodic removal of the dissolution medium for spectroscopic measurements. This opening must be at least 1 cm away from the wall of the dissolution vessel to collect dissolution solution for concentration monitoring.^34^ The dissolved drug concentration must be sampled in the bulk. The stirring speed was set to 100 rpm. After the naproxen sodium tablet was placed in the dissolution basket, the basket was capped to prevent solvent evaporation. Drug dissolution was started with stirring, and a timer was started for the kinetic recording. An aliquot of 5 mL of release medium was collected every 5 min in a 2 h period. The dissolution medium was replenished immediately with a 5 mL addition after each sample was taken. For each sample, 10 replicate absorbance values were recorded at a wavelength of 329 nm by LoggerPro (Vernier Software & Technology) with 5 s intervals. Absorbance of a naproxen sodium standard reference solution was measured every 30 min to monitor the instrumental drift in the background during the kinetic experiment. This is an important concept about kinetic measurements to teach the students: in a long kinetic run, the potential instrumental drift must be accounted for in accurate chemical measurements.

## Hazards

The experimental protocol requires naproxen sodium, the active ingredient of an NSAID, and the corresponding drug tablets. All waste generated in this experiment can be disposed of down the drain with an excess amount of tap water. The metal bars and dissolution baskets used in the experiment have slightly sharp edges and can potentially cause skin cuts. They should be handled with care.

## Implementation Setting

The experimental protocol was completed by students in an undergraduate lab class (CHEM:2021 Fundamentals of Chemical Measurements) for seven semesters for a total of 183 students, a high school student who attended a summer research program, and an undergraduate researcher. There was excellent consistency in student results across all seven semesters of the course. Fundamentals of Chemical Measurements is a laboratory course students take after completing two semesters of freshman chemistry and typically consists of up to 24 students per semester in the Spring (one section) and up to 48 students in the Fall (two sections). The class is taken by chemistry majors and students from other disciplines, including biochemistry, pharmacy, and engineering. In addition to building laboratory skills in chemical measurements, there is a strong emphasis on statistical analysis of data in the course, including accuracy and precision of experimental results, error distribution, error propagation, hypothesis testing, and regression analysis. Laboratory periods were 2 h and 50 min in length. Students were arranged into pairs and very occasionally groups of three to perform the dissolution experiment. The laboratory exercise requires two students to be able finish all measurement steps within each five min interval, with one student managing the kinetic aspect while the other student takes spectroscopy measurements. Each group of students performed two laboratory sessions of the dissolution experiment, switching their measurement roles between the 2 days. All students successfully built their dissolution apparatus within minutes and completed the measurements within their lab periods.

## Analysis of Dissolution Kinetics

### Students’ Dissolution Curves

**Figure 4:**
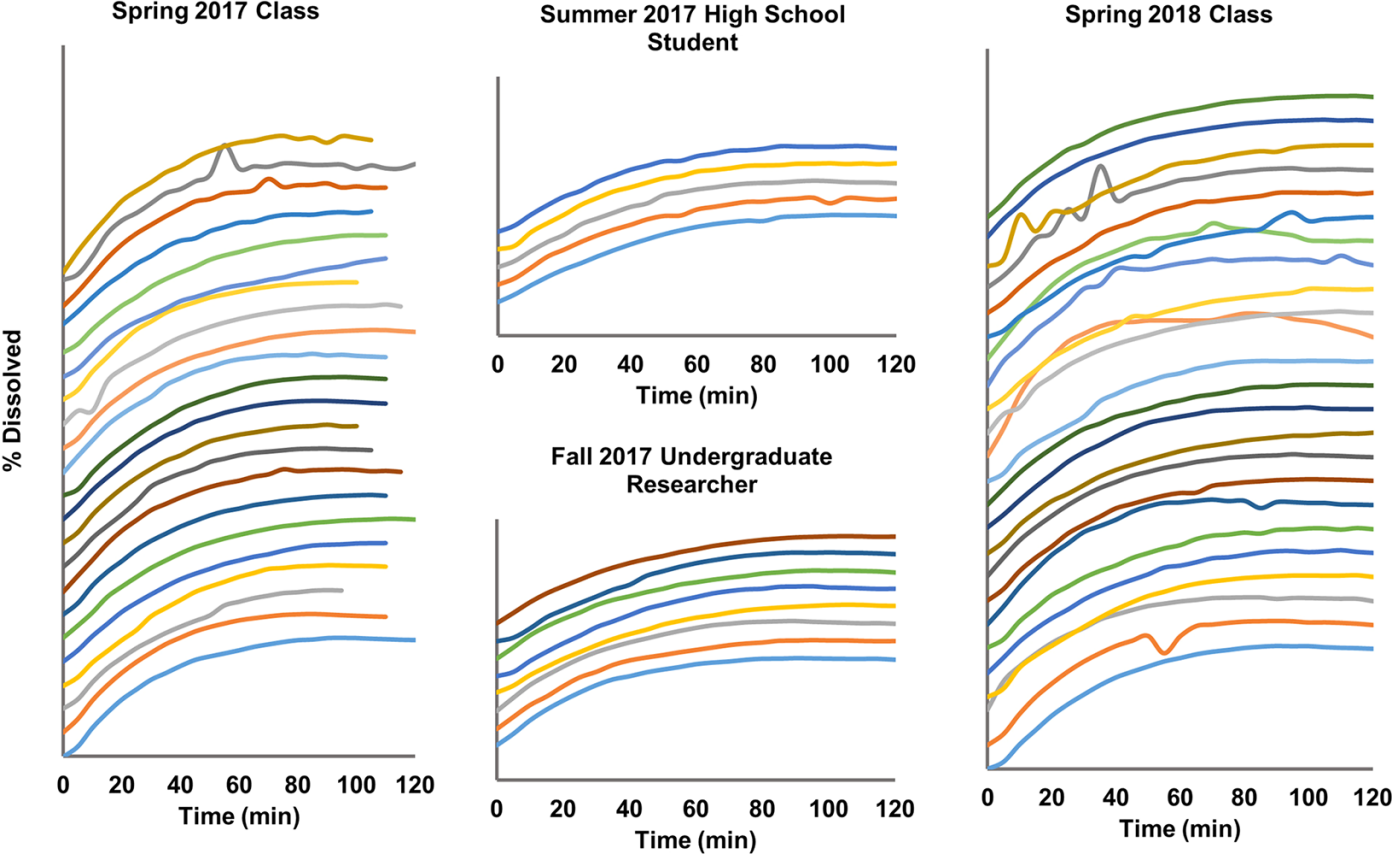
Dissolution curves generated by students in four sessions of different semesters. Each curve was plotted with the same scale, from 0% to 100% in percent dissolution, and was shifted upward to generate space between curves for clarity. A total of 59 curves were collected in these sessions.

The students’ dissolution curves presented here correspond to four sets of data: Spring 2017 Class, Summer 2017 Researcher, Fall 2017 Researcher, and Spring 2018 Class. Figure 4 shows all 59 dissolution curves that these students collected.

**Figure 5:**
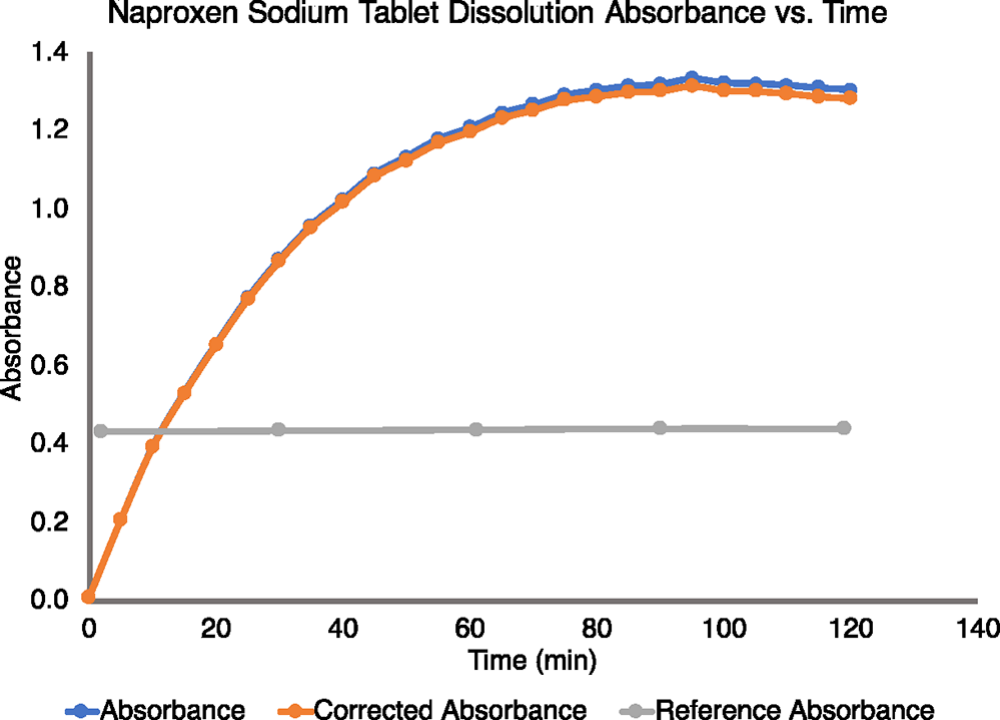
Dissolution profiles (absorbance vs time) of a naproxen sodium tablet before and after correction of instrument drift. The gray curve shows the absorbance of a naproxen sodium reference standard solution collected every half hour.

Students constructed the dissolution profiles of naproxen sodium tablets by plotting the absorbances at all dissolution time points. As the dissolution took place over a 2 h duration, absorbance of a reference solution was measured every 30 min to observe the spectral drift of the background. Students performed linear regression analysis and *t* test on the absorbance of the reference solution across the 2 h to determine if a correction procedure was required. Figure 5 shows a student’s dissolution curve before and after the correction, with the absorbance of the reference solution overlaying on the same plot. The results of linear regression and the *t* test are shown in Table 1. The *t* statistic of the slope was calculated to be 5.845, which was larger than the *t*-value of 3.182 at 95% confidence level, indicating that the slope was significant (*p* < 0.01) and an instrument drift occurred during the kinetic experiment. The kinetic curve thus needed to be corrected with this instrument drift.

**Table 1:** Output of Linear Regression Analysis from the Excel LINEST Function^a^

Slope	6 × 10^–05^	0.4335	*y*-Intercept
*u*_m_	1 × 10^–5^	0.0007	*u*_b_
*R*^2^	0.919	0.0009	*S*_*y*_
*F* statistic	34.2	3	df
Regress. ss	3 × 10^–5^	3 × 10^–6^	Residual ss

					
Slope	6 × 10^–5^	Intercept	0.4335	*r*^2^	0.919
Error	1 × 10^–5^	Error	0.0007		
*t*_calc_	5.845	*t*_calc_	578.674	*F*-value	34.162
*t*_95%_	3.182	*t*_95%_	3.182	*F*-critical	10.128
*p*	9.98 × 10^–3^	*p*	1.14 × 10^–8^	*p*	0.010
Significance	yes	Significance	yes	Significance	yes

a t test and goodness of fit results are listed in the table.

### Nonlinear Least Squares (NLLS) Analysis and Comparison of Dissolution Profiles

To further understand the dissolution data, students performed nonlinear least-squares (NLLS) analysis of the dissolution curves, applying mathematical models for drug dissolution and release. Peppas Law^35^ was selected for this laboratory protocol as it is a widely used model for analyzing drug dissolution and release processes. Peppas Law (eq 1) is used extensively to describe the first 60% fractional release of drug molecules,^35^ where the fraction of release *Q*_*t*_ at time *t* is
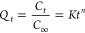
1where *C*_*t*_ is the concentration of released naproxen sodium at time *t* and *C*_∞_ is the concentration of released naproxen sodium at time infinity. *K* is the release constant related to the loading concentration and the diffusion coefficient; *n* is the release exponent determined by the geometry of the drug formulation and the release mechanism (Fickian diffusion controlled or non-Fickian). The dissolution law is applicable to the first 60% of drug dissolution. Students were directed to apply Excel Solver to complete the NLLS fitting process and obtain the kinetic parameters *K* and *n*. Students used the Excel Solver function to perform the fitting analysis, paying extra attention to test the possibilities of local minima in the χ^2^ surface and to ensure that the global minimum was reached for the correct kinetic parameters. The general concepts of NLLS were detailed in lectures as NLLS fitting is typically an advanced numerical analysis method in sophomore level laboratory courses. The Solver in Microsoft Excel^36^ is used for the NLLS analysis in this experiment because of the wide availability of the software.

**Figure 6:**
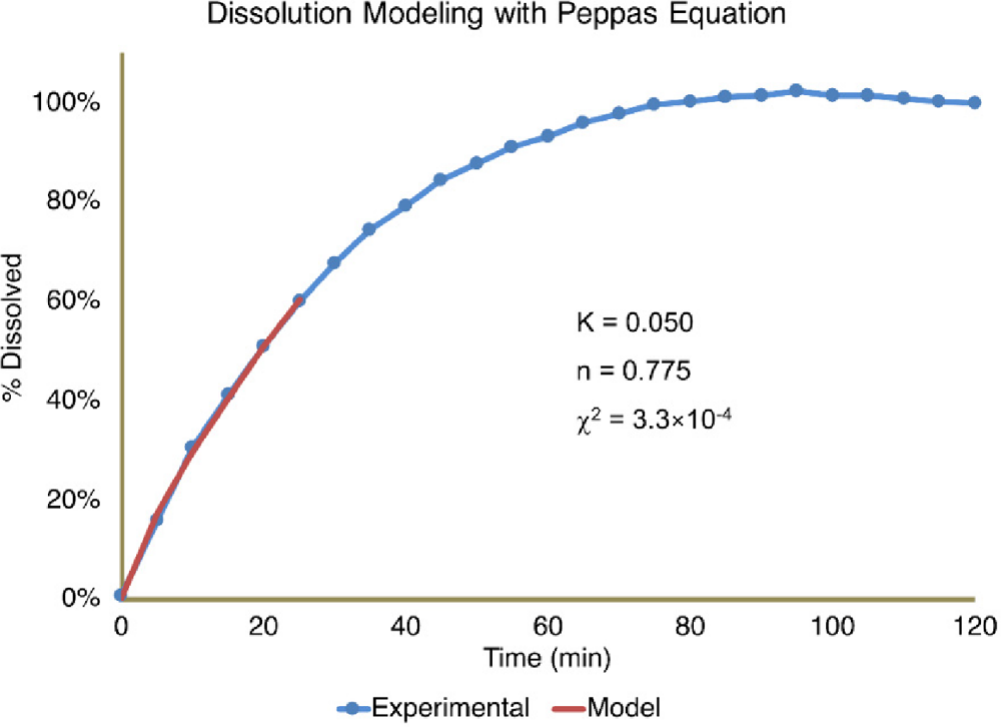
Representative nonlinear fitting of student kinetic data with Peppas Law.

Figure 6 shows a fitting result for a representative student data trace.

To test the effectiveness of the experiment protocol in teaching students the experimental skills in drug dissolution, we fitted all student kinetic curves with dissolution models to summarize and present student data in its entirety. We encountered a challenge that is specific and undoubtedly common in undergraduate lab courses, where students come into the lab course with different levels of lab skills. An example is the fourth curve from the top of the Spring 2018 student data in Figure 4. This kinetic profile shows significant variations in the data points, indicative of this student group’s possible lack of experience and precision in sample collection and spectroscopic measurements. We (1) discussed potential reasons for the large fluctuations with the class, including light scattering from microparticles formed by fillers in the tablets, and (2) developed a statistical protocol to detect outlier data points in the dissolution curves. These experimental factors that could potentially result in deviations and the outlier testing are detailed in the Section III of the Supporting Information. Student kinetic data files of drug dissolution showed excellent consistency with the Peppas law, as demonstrated by the good overlap between experimental data points and the mathematical model of dissolution, the small χ^2^ values of fitting (Table S1), and the randomness of the fitting residues.

Beyond the NLLS analysis of their data, students applied a model-independent method to compare the dissolution profiles of the two tablets in their experiments. A model-independent mathematical approach introduced by Moore and Flanner^33,37^ was given to students for their calculation. This approach is recommended by the United States Food and Drug Administration (FDA) as a standard method to test the consistency of drug dissolution kinetics.^7^Equation 2 shows the difference factor (*f*_1_), while eq 3 is the similarity factor (*f*_2_):
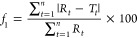
2
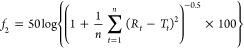
3where *R*_*t*_ is the percent dissolution of the reference drug formulation at time *t* and *T*_*t*_ is the percent dissolution of the test formulation at time *t*. For students to compare their dissolution profiles, they chose one of the curves they collected as the reference, while the other curve was selected as the test. FDA has set the standard thresholds of *f*_1_ smaller than 15 and *f*_2_ greater than 50, indicating consistency between two dissolution profiles.^7^

**Figure 7:**
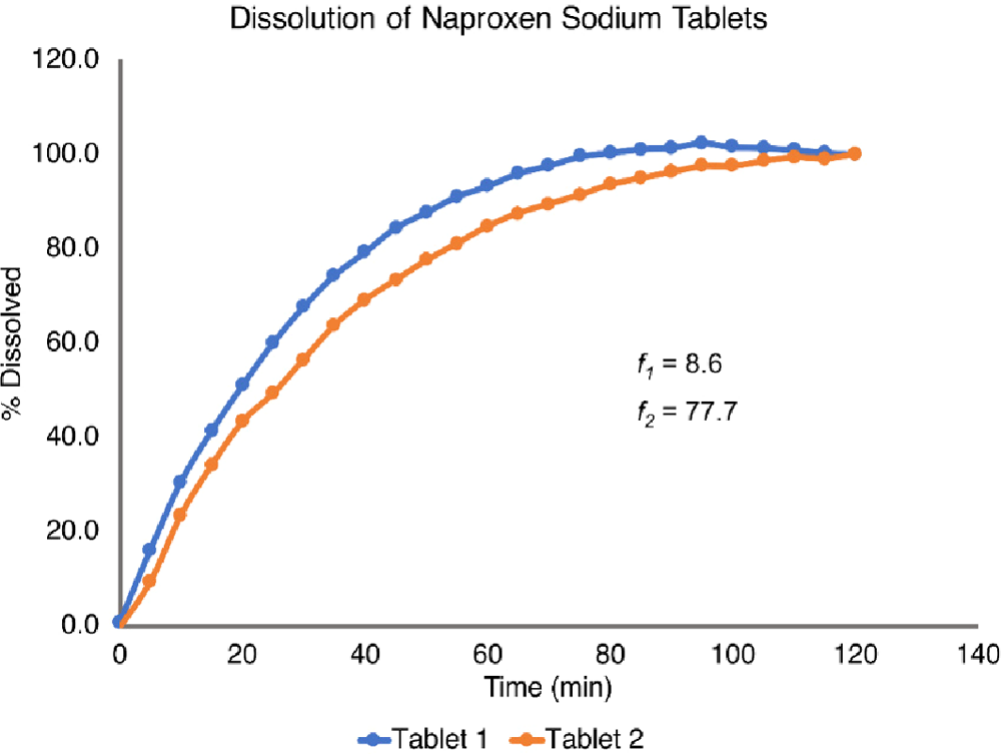
Comparison of the two trials of a student pair’s dissolution data with *f*_1_ and *f*_2_ methods.

Figure 7 shows two representative dissolution profiles with the calculated *f*_1_ and *f*_2_ values.

The calculated *f*_1_ = 8.6 and *f*_2_ = 77.7 values suggest that the two tablets show consistent dissolution kinetics as *f*_1_ < 15 and *f*_2_ > 50. From this analysis, students learned to compare their calculation results to the thresholds to assess the consistency of the dissolution profiles of the two tablets they tested. At the end of the laboratory session, students were able to apply the knowledge of pharmaceutical chemistry to perform the tests frequently done in the pharmaceutical industry.

## Assessments of Students’ Achievement of Learning Goals

Students’ achievements of the designated learning goals were assessed.

- Build a dissolution apparatus and perform dissolution testing, generating dissolution curves with UV spectrophotometry data.

During the dissolution laboratory sessions, two teaching assistants circulated through the lab and checked the dissolution devices that students constructed before they started the kinetic measurements. All students successfully constructed their dissolution apparatus, paying close attention to the accurate volume of dissolution solvent in the device determined by solvent mass, the sealing of the device top to avoid solvent evaporation during the lengthy kinetic experiment, and the sampling location to ensure that a homogenized dissolution solution is taken for concentration measurements. Students performed dissolution tests with their constructed apparatus by extracting dissolution solution samples at 5 min intervals and replenishing the dissolution solvent, assayed the samples with UV spectrophotometry following a comprehensive procedure for accurate measurements, and generated dissolution profiles from the absorbance data. In constructing the dissolution apparatus and making spectroscopy measurements, students practiced and worked on the concepts of accurate and precise measurements through an attention to experimental details. For example, in dissolution testing, the dissolution solution must be replenished for accurate concentration determination; in spectroscopy measurements, the cuvette must be rinsed with solvent and then the sample solution multiple times before spectroscopy measurements were conducted, and the same surface of the cuvette must face the light source in all measurements to minimize the effect of optical phenomena such as surface reflection and sample scattering. Furthermore, students practiced and gained experience with special considerations in kinetic measurements: for example, the solution must be sealed to avoid solvent evaporation and maintain accurate concentration, and spectroscopy measurements of a standard solution must be conducted concurrent to sample measurements to account for potential instrumental drift during the two-hour kinetic measurements. Students used hypothesis testing, including an *F* test of the linear regression of the standard solution kinetic data and *t* tests of the significance of the slope and intercept. If a drift was detected by a statistically significant slope, a correction for drift was introduced in the calculation to obtain the accurate dissolved concentrations in the kinetic curve.

- Use nonlinear least-squares (NLLS) analysis to fit the dissolution profiles with a widely used dissolution model.

In preparing lab reports, all students in the semesters this experiment has been implemented were able to perform NLLS analysis to fit their dissolution profiles with the Peppas kinetic model. They tested the effect of the stopping criteria on the iteration procedure, including the maximum number of iterations allowed and the change in chi square value Δχ^2^ between iterations. They repeated the fitting procedure multiple times until the χ^2^ value was constant to ensure that the minimum of the χ^2^ surface had been located. They performed the fitting procedure with different sets of initial parameter values to make sure that the fitting was not trapped in a local minimum and the global minimum of the χ^2^ surface had been reached. They examined the overlap between the fitting curve and the experimental kinetic data and the randomness of the residual plot to assess the goodness of NLLS fitting. In generating lab reports, students practiced concepts of nonlinear fitting, for example, why an iterative process is needed in nonlinear fitting while not in linear regression, the statistical characteristics of a good fitting in NLLS, what are local minima, and how to ensure the global minimum had been reached for correct fitting.

- Evaluate the consistency in dissolution profiles per FDA regulated criteria.

Another important learning goal for students is to become familiar with the FDA regulated criteria for comparing the dissolution profiles. In the most recent implementation of the experiment, the Fall semester of 2023, student lab reports showed that all students were able to compare the dissolution profiles generated in the dissolution session with the FDA regulated model-independent method. By calculating the *f*_1_ and *f*_2_ factors, they were able to evaluate the level of similarity and difference between the dissolution profiles using the FDA criteria.

- Have an opportunity to experience protocols related to pharmaceutical research and development.

Through this new drug dissolution experiment, students had an opportunity to experience a central concept and experiment in pharmaceutical science: the monitoring of drug dissolution kinetics. Student outcomes show that they acquired an excellent understanding of the experimental procedures, complex analysis of the kinetic data, and the FDA guidelines in pharmaceutical research and development.

Toward the end of the Spring 2017 semester, students were given an assignment to perform a literature review of a research article in a field that they were interested in (Section V of the Supporting Information). Students were given significant flexibility to select a topic in any area of chemistry, biology, medicine, or engineering. After selecting a research article, students were guided to summarize the goals, methodologies, and results, express their evaluations, and provide future directions of the research. Thirteen out of twenty-three students selected an article in the research field of drug delivery, which showed many students had developed an interest in pharmaceutical chemistry, particularly in drug delivery related topics, after completion of the dissolution laboratory.

## Conclusion

A dissolution protocol has been developed for undergraduate students to build a dissolution apparatus, monitor drug dissolution kinetics, perform NLLS analysis of the dissolution data to extract kinetic parameters, and compare the dissolution profiles by applying USP mandated methods. The protocol was evaluated to be able to generate reproducible dissolution data by different groups of experimenters over a large time span and at various experiment locations. The protocol has been utilized successfully in seven semesters and will be continually implemented in the undergraduate laboratory teaching curriculum.

## Supporting Information Available

Dissolution apparatus, optimization of the experimental conditions for spectroscopic monitoring, summary of student data, lab manual, and end of semester paper (PDF; DOCX)
Dissolution curve and modeling data (XLSX)
