# Supplementary material for: Spectroscopic Monitoring and Modeling Drug Dissolution for Undergraduate Chemistry Curriculum
Source: J Chem Educ. 2024 Mar 12;101(4):1648–55. doi: 10.1021/acs.jchemed.2c00707 (PMC11008090; doi:10.1021/acs.jchemed.2c00707)
Supplement: Supplementary file 2 — ed2c00707_si_002.docx [file ed2c00707_si_002.docx]

**Supporting Information**

**Spectroscopic Monitoring and Modeling Drug Dissolution for Undergraduate Chemistry Curriculum**

Chengxuan Guo^1^, Nicole Wendel^1^, Ally Lee^2^, Shonda Monette^1^, Brian Morrison^1^, Dominic Frisbie^1^, Earlene Erbe^1^, Renée S. Cole^1*^, Max Lei Geng^1*^

^1^Department of Chemistry, University of Iowa, Iowa City, IA 52242

^2^Chadwick International School, 45, Art center-daero 97 beon-gil, Yeonsu-gu, Incheon, 22002 South Korea

[*renee-cole@uiowa.edu](mailto:*renee-cole@uiowa.edu)

*[lei-geng@uiowa.edu](mailto:lei-geng@uiowa.edu)

**Supporting Information**

**Table of Contents**

| I. Dissolution Apparatus | 3 |
| --- | --- |
| II. Optimization of the Experimental Conditions for Spectroscopic Monitoring | 5 |
| III. Summary of Student Data | 8 |
| IV. Lab Manual | 11 |
| V. End of Semester Paper: Review of a Research Article | 19 |

**Supporting Information**

**I. Dissolution Apparatus**


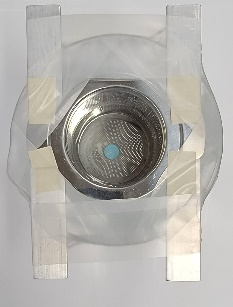

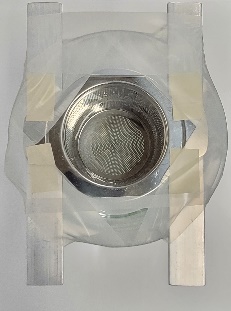

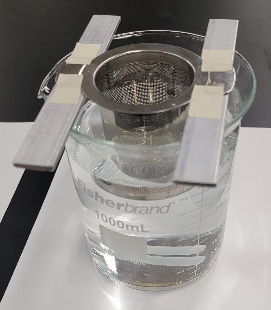

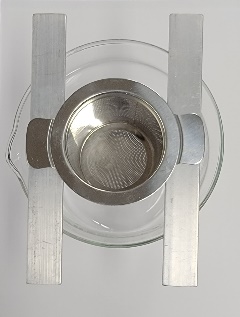

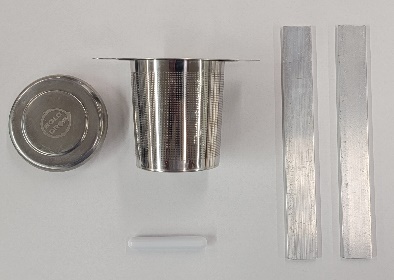


**B**

**A**

**C**

**D**

**E**

Figure S1. Set up of the dissolution apparatus.

1. Components of the set up: dissolution basket and the lid, supporting aluminum bars and a 2-inch stirring bar. The aluminum bars were *6"L× 34" W× 18" H* in dimension and were prepared by the Department of Chemistry machine shop. They were cut from *48"L× 34" W× 18" H* aluminum pieces that can be acquired from any construction shops. The aluminum bars were polished to remove all sharp edges. Glass rods were tested as supporting bars in the initial experiment development and were found to be too slippery to securely support the dissolution basket. The large 2-inch stirring bars were selected to generate sufficient force to mix the 900-mL dissolution solution.
2. The dissolution basket was supported with two aluminum bars and centered onto a 1 L beaker as the dissolution vessel.
3. The basket was secured to the supporting bars. Dissolution solution of 900 mL in volume was added into the beaker, and the volume was calibrated with mass at the recorded room temperature.
4. The top of the dissolution apparatus was sealed with parafilm and an opening was left (at the bottom of the image) for withdrawing solution in kinetic measurements.
5. A naproxen sodium tablet was placed at the center of the dissolution basket, the basket was covered tightly with a lid and the dissolution experiment started with stirring. The parafilm piece at the bottom was used to seal the opening between solution withdraws.

For instructors in need of the aluminum bars for the experiment (for free), please contact the corresponding authors of the article.

Many alternative infuser baskets are available to be used for the experiment, including the ones from the following sites, at a cost of ~ $10-15. These baskets share the shape and properties of the one used in the experiments described in the manuscript. The structural rigidity of the basket should be a top consideration in the selection of baskets, with stainless steel as a choice material. The wings on the side facilitate the assembly of the dissolution structure and the cover helps to prevent solvent evaporation during the kinetic measurements.

<https://www.oxo.com/oxo-tea-infuser-basket.html?queryID=4cafaf7de7ff64b58fb4c142fbc45055&objectID=5344&indexName=magento2_prod_oxooxo_products>

<https://www.amazon.com/Yoassi-Extra-Fine-Approved-Stainless/dp/B01LQ7NQTW/ref=pd_bxgy_sccl_2/142-5209728-0395936?pd_rd_w=YZpO6&content-id=amzn1.sym.26a5c67f-1a30-486b-bb90-b523ad38d5a0&pf_rd_p=26a5c67f-1a30-486b-bb90-b523ad38d5a0&pf_rd_r=5E53PA4CVRWT3MWC8KAN&pd_rd_wg=iwykW&pd_rd_r=86f083e0-b36e-453d-8b8a-292ddbde5b8c&pd_rd_i=B01LQ7NQTW&th=1>

<https://www.amazon.com/dp/B0BFHHJYBC/ref=sspa_dk_detail_6?ie=UTF8&pd_rd_i=B0BFHHJYBCp13NParams&s=kitchen&sp_csd=d2lkZ2V0TmFtZT1zcF9kZXRhaWxfdGhlbWF0aWM&th=1>

Student experience in this experiment prepares them for drug dissolution measurements in their future careers with USP dissolution devices and instruments, for example, with an Agilent dissolution basket (Figure 2, Dissolution Baskets, Part Number:12-2100, 40-Mesh, 381 µm, SST, cost $188, <https://www.agilent.com/store/en_US/Prod-12-2100/12-2100>, assessed on February 21, 2024), with an USP dissolution apparatus (Agilent 708-DS Dissolution Apparatus, 6-position, cost ~ $30,000).

**Supporting Information**

**II. Optimization of the Experimental Conditions for Spectroscopic Monitoring**

Spectroscopic monitoring of the dissolution of naproxen sodium tablets was performed by measuring the dissolution medium at sequential time points with a portable USB4000 UV/Vis Spectrophotometer (Ocean Optics, Inc., Largo, FL). To develop the protocol of the dissolution measurements, experimental conditions needed to be properly selected to construct a dissolution curve with an endpoint absorbance that is within the optimal absorbance range (A < 1.5), and the completion time of the dissolution should be adjusted to fit the undergraduate laboratory sessions. The key experimental parameters included the absorption maximum (λ_max_) at which the dissolution is monitored, amount of naproxen sodium molecules in the tablet, and stirring speed. A series of experiments were conducted to determine these experimental parameters. Figure 3 shows the UV-vis absorption spectrum of naproxen sodium solution at a concentration of 0.15 mM. The spectrum consists of four absorption peaks at 262 nm, 271 nm, 315 nm, and 330 nm. The absorption maximum around 271 nm was chosen to assay the standard naproxen sodium solutions to obtain the molar absorptivity at the absorption maximum, according to the wavelength specified in the U.S. Pharmacopeia protocol for naproxen sodium,^1^ and the absorption maximum around 330 nm was selected for the dissolution test according to USP dissolution protocol of naproxen sodium tablets.

A set of preliminary tests were performed by monitoring the dissolution processes at different trial conditions of varying tablet sizes and stirring speeds. The preliminary experiments were done to compare the dissolution time to the laboratory session length. The details of these tests are included in the supplemental information. A final trial was conducted closely following the USP dissolution monograph of naproxen sodium tablets, by dissolving a full tablet at a slow stirring speed of 60 rpm. Solution absorbance was measured at 329.3 nm, an absorption peak with a lower molar extinction coefficient (ε = 1.4×10^3^ M^-1^cm^-1^). The dissolution completed in approximately 80 min, meeting the expectation of an optimal laboratory time close to 1.5 hrs. The solution absorbance was ~ 1.4 when the tablet was completely dissolved, a value that is appropriate for absorption measurements.

The key experimental parameters were optimized for the dissolution protocol. The USP dissolution protocol for naproxen sodium tablets defines the paddle rotating speed to be 50 rpm using apparatus 2. However, stir plates were utilized in this experimental design, the minimum stirring speed available on the stir plate is 60 rpm, which was observed to stir quite slowly. In the students’ experiments, the stirring speed was slightly increased to 100 rpm to ensure sufficient stirring power for mixing of the dissolution medium. Figure S2 shows the difference between the dissolution curves at 60 rpm and 100 rpm. The dissolution curve collected at a stirring speed of 100 rpm shows a faster increase in percent dissolved, however, there is no drastic change in the dissolution completion time.

Figure S2: Dissolution curves at stirring speed of 60 rpm of the development trial experiment and 100 rpm in one student’s dissolution representative dissolution curve.

Figure S3: Dissolution curves generated using standard USP Apparatus 2 (paddle model) and using the designed apparatus.

A comparison of the naproxen sodium reference dissolution curve generated using standard USP Apparatus 2 from a literature article^2^ and the designed apparatus is shown in Figure S3. The dissolution is faster when using standard USP Apparatus 2 and completes within 45 minutes, where dissolution using the designed apparatus completes within 1.5 hrs. The designed apparatus generates a slower dissolution profile with the dimensions and structures of the tea infuser customized dissolution basket and the magnetic stir bar/stir plate set-up.

In this drug dissolution experiment, we discuss that dissolution monitoring in pharmaceutical laboratories uses one of the seven designs of dissolution apparatuses authorized by the USP, that all components in the dissolution devices are manufactured with stringent dimensions specified by the USP, and that exacting conditions must be followed in drug dissolution monitoring in order to guarantee consistency between laboratories across the world. We discuss that while the kinetic parameters measured by our experimental setup will be different from those measured with USP dissolution apparatuses, our experimental results will still have high degrees of consistency and reproducibility. Through this process, students learn that a calibration procedure can theoretically correlate our kinetic parameters to those measured by USP devices, continuing our discussion of experimental accuracy throughout the semester. The experiment allows students an opportunity to gain a deep appreciation of the concept of in-vitro in-vivo correlation, a central concept in pharmaceutical sciences, where the kinetic rates measured in a dissolution device in a laboratory can be used to predict the drug dissolution rates in human body once a correlation is established.

**Supporting Information**

**III. Summary of Student Data**

To test the effectiveness of the experiment protocol in teaching students the experiment skills in drug dissolution, we fitted all student kinetic curves with dissolution models to summarize and present student data in its entirety. We encountered a challenge that is specific and undoubtedly common in lab courses where students come into the lab course with different levels of lab skills. An example is the fourth curve from the top of the Spring 2018 student data in Figure 3. This kinetic profile shows significant variations in the data points, indicative of this student group’s possible lack of experience and precision in sample collection and spectroscopic measurements. We discussed some potential reasons for the large fluctuations with the class. For example, between two spectroscopy measurements, the experimental protocol calls for the rinsing of the cuvette with the dissolution solvent three times and then the sample solution three times, followed by rinsing the outside walls of the cuvette with water and drying. If any water entered the cuvette during the final step, the apparent concentration, and thus the absorbance, would drop for that data point in the kinetic trace. Sudden increases in the absorbance values could be the result of small particles crossing the beam path. Each drug tablet used in the experiment weighs ~ 300 mg, varying in weight slightly from tablet to tablet, and contains 220 mg of naproxen sodium per the manufacturer. The rest of the weight was the inactive ingredients of the tablet, including glycitols and polymer fillers. Some of these inactive ingredients stayed in the solution as microcrystalline colloidal particulates; at the end of dissolution, the solution became milky cloudy. In the initial development of the experiment, we tested a number of filtering methods, including filter papers of different pore sizes and syringe filters with nanometer pores, to remove these microparticles before the spectroscopy measurements. We discovered that it was very unlikely for undergraduate students to complete all these experimental steps during the five-minute sampling interval, and that the microparticles did not alter the absorbance values significantly. The kinetics of dissolution can be accurately monitored without solution filtering. A compromise is, however, that the occasional larger particles in the solution caused by the occasional fracturing of the tablet, can cross the light beam during the five-second absorption measurement, resulting in sudden increases in the absorbance, and thus large fluctuations in the kinetic curve. This calls into question the validity of the data traces. We set out to establish a method to test outliers in student data in nonlinear least squares fitting. Although the outlier detection protocol we developed can be used to remove the outlier data points in a kinetic data trace and allow the NLLS fitting the rest of the data, we decided to apply a more rigorous measure: once an outlier data point is confirmed in the dissolution curve, the dissolution curve with the outlier is considered as an outlier curve to be discarded from further analysis. The rationale is that if a student group makes a solution and/or a measurement that produces an absorbance that is 30% off the expected value, the validity of the entire kinetic curve is in question. For the students’ dissolution data, 12 out of 59 curves were discarded applying the studentized residual method, one incomplete curve was removed, with the remaining 47 curves moved forward for nonlinear regression analysis and the evaluation of consistency.

Table 2 lists the kinetic parameters and χ^2^ values obtained from the NLLS fitting by students. The parameters for curves with outlier data points are excluded from the table. The parameter *K*, associated with loading concentration in the tablet and the diffusion constant of the drug molecules, are calculated to be between 0.02 to 0.15, with the majority of the numbers to be in the range of 0.02-0.04. The Spring 2018 Class has five out of eighteen *K’*s that fall outside of the range 0.02-0.04, whereas the results of other student sets fall consistently within the range of 0.02-0.04.

**Table S1: dissolution parameters obtained from nonlinear regression analysis of student data.**

|  | **Spring 2017 Class** | | | **Summer 2017 High School Student** | | | **Fall 2017 Undergraduate Researcher** | | | **Spring 2018 Class** | | |
| --- | --- | --- | --- | --- | --- | --- | --- | --- | --- | --- | --- | --- |
| Curve Number | K | n | χ^2^ | K | n | χ^2^ | K | n | χ^2^ | K | n | χ^2^ |
| 1 | 0.02 | 0.99 | 2x10^-3^ | 0.02 | 0.92 | 8x10^-4^ | 0.04 | 0.86 | 7x10^-4^ | 0.02 | 1.04 | 3x10^-3^ |
| 2 | 0.03 | 0.90 | 1x10^-3^ | 0.02 | 0.98 | 2x10^-3^ | 0.04 | 0.85 | 9x10^-4^ | 0.15 | 0.49 | 9x10^-3^ |
| 3 | 0.03 | 0.89 | 2x10^-3^ | 0.02 | 1.00 | 4x10^-3^ | 0.04 | 0.81 | 4x10^-4^ | 0.03 | 0.89 | 4x10^-3^ |
| 4 | 0.02 | 1.05 | 7x10^-4^ | 0.02 | 0.93 | 2x10^-3^ | 0.03 | 0.90 | 8x10^-4^ | 0.04 | 0.79 | 7x10^-4^ |
| 5 | 0.03 | 0.86 | 1x10^-3^ |  |  |  | 0.02 | 1.03 | 2x10^-3^ | 0.03 | 0.90 | 3x10^-3^ |
| 6 | 0.03 | 0.85 | 2x10^-3^ |  |  |  | 0.04 | 0.79 | 8x10^-4^ | 0.04 | 0.92 | 3x10^-4^ |
| 7 | 0.03 | 0.90 | 2x10^-3^ |  |  |  | 0.02 | 0.99 | 4x10^-3^ | 0.03 | 0.90 | 1x10^-3^ |
| 8 | 0.03 | 0.91 | 8x10^-4^ |  |  |  | 0.04 | 0.78 | 7x10^-4^ | 0.05 | 0.77 | 3x10^-4^ |
| 9 | 0.04 | 0.84 | 2x10^-3^ |  |  |  |  |  |  | 0.03 | 0.84 | 2x10^-3^ |
| 10 | 0.03 | 0.91 | 2x10^-3^ |  |  |  |  |  |  | 0.03 | 0.91 | 5x10^-4^ |
| 11 | 0.03 | 0.89 | 6x10^-4^ |  |  |  |  |  |  | 0.04 | 0.80 | 5x10^-4^ |
| 12 | 0.02 | 0.98 | 4x10^-3^ |  |  |  |  |  |  | 0.02 | 0.95 | 4x10^-3^ |
| 13 | 0.04 | 0.80 | 2x10^-3^ |  |  |  |  |  |  | 0.06 | 0.95 | 1x10^-3^ |
| 14 | 0.03 | 0.92 | 2x10^-3^ |  |  |  |  |  |  | 0.03 | 0.86 | 3x10^-3^ |
| 15 | 0.02 | 1.02 | 1x10^-3^ |  |  |  |  |  |  | 0.06 | 0.70 | 2x10^-3^ |
| 16 | 0.02 | 0.91 | 3x10^-3^ |  |  |  |  |  |  | 0.04 | 0.81 | 2x10^-3^ |
| 17 | 0.02 | 0.98 | 1x10^-3^ |  |  |  |  |  |  | 0.03 | 0.89 | 2x10^-3^ |
| 18 |  |  |  |  |  |  |  |  |  | 0.05 | 0.87 | 3x10^-4^ |
|  |  |  |  |  |  |  |  |  |  |  |  |  |
|  |  |  |  |  |  |  |  |  |  |  |  |  |

**Supporting Information**

**IV. Lab Manual**

**Experiment 6: Modeling Dissolution of Naproxen Tablets**

**INTRODUCTION**

In this experiment, we will monitor the dissolution of naproxen tablets with molecular absorption spectrophotometry and model the kinetics. The timed release of drug molecules from the tablets will be recorded to yield the kinetics of the release process. Modeling of the kinetics provides information on the mechanism of the release.

The development of new drug formulations and novel delivery vehicles to achieve effective and targeted drug delivery is one of the essential tasks in pharmaceutical research. It is a central goal actively pursued in the field of nanotechnology and nanomedicine today. Monitoring the release process helps guide the structural design of the delivery vehicles to effectively control the release process. The advancement in nanotechnology in recent years has opened up tremendous opportunities in novel drug release vehicles, and dissolution monitoring has played a valuable role in these developments.

Drug dissolution is commonly monitored with the apparatuses developed by the US Pharmacopeia, with standardized equipment and methods for specific drug formulations. These apparatuses generate efficient agitation and mixing of the release medium to facilitate good contact between the drug formulation and the medium, to allow accurate recording of the dissolution. With the rapid development of new delivery vehicles, for example in nanotechnology, new procedures are being devised to monitor drug release. In pharmaceutical industry, dissolution monitoring is used in quality control to ensure consistency between batches and that new formulations are consistent with reference standards. Dissolution tests supply a potential substitute for human studies when the correlation between *in vitro* dissolution data and *in vivo* drug absorption (in-intro in-vivo correlation, IVIVC) is established.

We will monitor the dissolution of the naproxen sodium tablets and the release of the drug molecules into the medium in this experiment. The measurement of the solution absorbance in the ultraviolet region over time follows the release kinetics. To understand the fundamental processes that govern the kinetics, we will fit the release data to drug dissolution models to extract kinetic constants. We will learn the nonlinear least squares (NLLS) method to model experimental data to nonlinear functions.

The kinetics of drug release is controlled by the geometry, composition and structure of the delivery vehicle, and the diffusion of drug molecules. The solution of Fickian diffusion equations yields specific results for different geometries of the formulation. In the initial part of the release process, typically in the first 60% of the release, the diffusion results can be approximated by simpler mathematical functions. In this lab, we will fit our data with the Power Law of drug release, where the fraction of release $Q_{t}$ at time $t$is

$$Q_{t}=\frac{C_{t}}{C_{\infty}}=Kt^{n}$$

In this equation, $C_{t}$ is the concentration of released drug at time *t*, $C_{\infty}$ the concentration of released drug at time infinity (at long time), $K$ the release constant related to the loading concentration and the diffusion coefficient, and 𝑛 the exponent related to the geometry of the formulation and the release mechanism.

To model experiment data to a nonlinear function, nonlinear least squares regression is used, by minimizing the chi-squared value $\chi^{2}$:

$\chi^{2}=\sum_{i=1}^{N} \left( Q_{i}-Q_{i, model} \right)^{2}$.

In this expression, $N$ is the number of data points used in the fit, $Q_{i}$ the experimental fractional release at time point 𝑖 and $Q_{i, model}$ the fractional release at time point 𝑖 estimated by the model. You will need to add Solver to Excel in order to perform the minimization, if it is not in Excel already. The details of the NLLS method and the minimization procedure will be discussed in lecture and we will practice the NLLS fitting in the second lab period.

The dissolution tests are used in pharmaceutical industry to assess the consistency between a test formulation that is being developed and a standard. The dissolution curves of the test and standard formulations are experimentally monitored, and then compared with the difference factor $f_{1}$ and the similarity factor$f_{2}$. A difference factor below 15 and a similarity factor above 50 are considered to indicate good consistency between the two samples being tested in dissolution studies. We will collect two dissolution profiles of the naproxen sodium tablets in the experiment and test their consistency using these two parameters.

**EXPERIMENTAL - you will perform this experiment in groups of two.**

**A. Spectrometer Setup**

1. Power up the Ocean Optics USB spectrophotometer by plugging the power adapter into one of the electrical outlets on the lab bench and connecting it to the computer via the USB cable and launching the Logger Pro software.
2. Obtain a quartz cuvette from your TA for the absorption measurements in the Ocean Optics spectrophotometer. A quartz cuvette is used in order to transmit the ultraviolet (UV) wavelength region where the naproxen absorption peaks are located.
3. To accurately account for optical effects such as reflection, always use the same cuvette at the same optical geometry for the blank and sample measurements.
   Mark one corner of the cuvette such that you always insert it into the sample compartment with the same orientation. Check to make sure that the cuvette is oriented such that the 1 cm path length is aligned with the beam path through the sample. Use the same cuvette for all measurements.
4. *Allow at least 20 minutes for the system to warm up and stabilize*.

**B. Preparation of Naproxen Stock Solution**

1. Naproxen sodium stock solution: Prepare a solution of approximately 2.5 x 10^-3^ M naproxen sodium in water by accurately weighing ~ 0.6400 g of naproxen and quantitatively transferring the solid to a 1 L volumetric flask. Dissolve in and dilute to approximately 3/4 volume with Millipore water. Mix the contents of the flask until all of the naproxen is dissolved. Dilute to the mark with Millipore water and mixing well by inversion.

**C. Preparation of Naproxen Reference Solution**

***Note: The solution prepared will be used on both days. Save it!***

1. Collect ~ 15 mL of the naproxen stock solution from your TA. Record the mass used in the preparation of the stock solution.
2. Reference solution: A reference solution of naproxen sodium is prepared to monitor the potential drift in the measurements during the timed kinetic measurements. Prepare the reference solution by quantitatively pipetting 12 mL of the naproxen stock solution into a 100 mL volumetric flask. Dilute to the mark with room temperature Millipore water and mix well by inversion.
3. Blank solution: Room temperature Millipore water will be used as the blank for absorption measurements.
4. Dissolution medium: Room temperature Millipore water will be used as the dissolution medium for drug release from the tablets.

**D. The Dissolution Apparatus**

1. Obtain a dry, 1 L beaker, a dissolution basket, two aluminum supporting bars and a large magnetic stirring bar from your TA.
2. Record the room temperature.
3. Place the magnetic stirring bar into the beaker. Place the dissolution basket on the rim of the beaker, supported by the two aluminum bars. Align the vertical axis of the basket with the center of the beaker. Secure the position of the basket with tape, verifying the two centers are within 5 mm of each other.
4. Weigh the beaker and record the mass.

**During the assembly of the dissolution apparatus (Steps 5-8), be careful not to splash any water out of the beaker. If any water spill occurs, the entire procedure must be restarted from Step 1.**

1. Using the mass difference (Mass of beaker and water – Mass of beaker) as the guide, quantitatively fill 900 g of Millipore water into the beaker. Be careful that no water is spilled onto the outside wall of the beaker or the scale during the measurement. Use a graduated cylinder to add water to ~ 890 g, then carefully add to 900 g using a plastic transfer pipet. Record the final mass of the beaker with water.
2. Set the 1 L beaker at the center of a magnetic stirrer; using a glass rod, carefully move the stirring bar to the center of the beaker.
3. Using parafilm, cover the top of the beaker to prevent evaporation during the dissolution experiment. Leave an opening on the side, between the rim of the basket and the wall of the beaker, for removing the dissolution medium. This opening should be at least an inch away from the beaker wall.
4. Turn on the magnetic stirrer, and increase the stirring speed to 100 rpm. Observe the stirring for a few minutes; smooth stirring is expected without causing the movement or wobbling of the beaker. When satisfactory, turn off the stirrer.

**E. Determination of the Wavelength of Observation**

1. Collect a blank spectrum and automatically subtract the blank from all subsequent collected spectra.
   1. Add ~3 mL of the calibration blank to your cuvette.
   2. Wipe down the sides of your cuvette with a clean Kimwipe, and place your cuvette in the spectrometer.
   3. Click on the Experiment menu 🡪 Calibrate 🡪 Spectrometer 1.

*NOTE: If the spectrometer has been on for at least 20 minutes, the warm-up step can be skipped.*

- 1. Click the “Finish Calibration” button and then “OK.”

1. Obtain a full spectrum of the reference solution and determine the wavelength of maximum absorbance.
   1. Rinse the cuvette three times with ~0.5 mL of the reference solution; then add ~3 mL of the solution to the cuvette.
   2. Use wash bottle to rinse the sides of the cuvette. Be careful not to get any water into the cuvette during washing. Wipe down the sides of your cuvette with a clean Kimwipe, and place your cuvette in the spectrometer.
   3. Click the green “Collect” button. Stop after 10 seconds.
   4. Scroll through the spectral data to identify the wavelength that corresponds to the maximum absorbance near 330 nm. Note that naproxen has a number of peaks in its absorption spectrum. This is the peak with the longest wavelength and will be used for the rest of the experiment. Record this wavelength (*λ_max_*) in your notebook (and lab report). *Consult your TA if this is not in the region near 330 nm (not more than 2 nm away from 330).*
   5. Copy and paste the absorbance measurements as a function of wavelength into the “UV-vis Data” worksheet of your experiment template.

**F. Dissolution of the Naproxen Sodium Tablet 1**

1. Obtain a naproxen sodium tablet from your TA; keep it in a small weighing boat. Do not handle the tablets with your hands, even when gloved.
2. Obtain 25 collection bottles from your TA. Label them with the collection times of 0, 5, 10, 15, …, 120 min, at 5 minute intervals. Arrange them in order to prepare for solution storage.
3. In the Dissolution Tablet 1 sheet of your Excel template, set up the calculation for the mean absorbance and plot the mean as a function of time, using scatter plot with straight lines connecting the data points. For convenience, you can keep the plot in the data sheet for now, and move it to Absorbance Plot 1 during lab report preparation. The plot will show zero absorbance across all time points, and the curve will be updated once you record absorbance data during the dissolution.
4. Reconfigure the spectrometer to collect time-based data at the wavelength of maximum absorbance that you determined (*λ_max_*). Set the instrument for recording 10 absorbance measurements for each solution in **5 second** increments.
5. Place a piece of folded weighing paper on the analytical balance and tare the balance. Directly transfer a tablet onto the weighing paper from the boat. Accurately weigh the naproxen sodium tablet; record the mass.

(Step 6 starts the kinetic measurements. Have the stop watch, micropipette, and all collection bottles ready before you start. Note that this is a kinetic experiment, so the removal of the release medium and the replenishment of the solution should be performed **quickly**.)

1. With weighing paper, carefully introduce the tablet to the center of the dissolution basket, ensuring that no water is splashed out of the beaker, and allow the tablet to sink to the bottom of the basket.
2. Immediately remove 5 mL of the dissolution medium with a micropipette and deposit it into the collection bottle labeled with “0 min”. Replenish 5 mL of the dissolution medium into the beaker. It is crucial to replace the dissolution medium; the results would be in error if this were not done.
3. Once the solution is replenished, immediately start the magnetic stirrer at the speed of 100 rpm.
4. Start the stop watch; this is the time zero (0 min) of the dissolution process.
5. Measure the absorbance of the release solution in the 0 min bottle at your wavelength of maximum absorbance (*λ_max_*). Record 10 absorbance measurements in 5 second increments.
   1. Pay careful attention to avoid carry-over of solution in the cuvette as you change samples. Rinse the cuvette with water for three times. (Note that in the next step you will use a **small** amount of the solution to rinse the cuvette each time. You will need over 2 mL of solution to make the absorbance measurements.) Then rinse the cuvette three times with the next solution to be measured.
   2. Rinse the outsides of the cuvette with water using a wash bottle. Be careful not to get any water into the cuvette during washing. Wipe the outside of the cuvette with a clean Kimwipe.
   3. Ensure that there are no bubbles in the cuvette before placing it back into the sample chamber.
   4. Record the ten absorbance values into your worksheet.
6. Measure the absorbance of the reference solution at *λ_max_*. Make 10 absorbance measurements in 5 second increment; record the exact time when the measurements are started.
7. At exactly 5 minutes after the previous solution was collected, remove 5 mL of the dissolution medium with a micropipette and deposit it into the next collection bottle.
8. Replace 5 mL of Millipore water into the beaker.
9. Measure the absorbance of the release solution that you just collected at your wavelength of maximum absorbance (*λ_max_*). Record 10 absorbance measurements in 5 second increments.
10. Repeat the release solution measurements (Steps 12-14) every 5 minutes, until 120 minutes or after the drug release curve has leveled off. Watch the release plot that you set up in Step 2 to determine if the dissolution has reached a plateau. Double check in the basket that the tablet has been completely dissolved. (Read Step 16 for the times of the reference measurements.)
11. At every 30 minutes (at 0, 30, 60, 90 and 120 min), measure the absorbance of the reference solution at *λ_max_*. Make 10 absorbance measurements in 5 second increment; record the exact time when the measurements are started.
12. Clean the 1 L beaker and the release basket with Millipore water and leave them to dry for experiment on Day 2.

(This is the end of experiment for Day 1.)

**G. Dissolution of the Naproxen Sodium Tablet 2 (Day 2)**

1. Start the Ocean Optics spectrophotometer and let it warm up for 20 minutes.
2. Repeat the dissolution measurements for the second naproxen sodium tablet. Record experiment data into sheet Dissolution Tablet 2.

**WASTE HANDLING**

All waste generated in this experiment can be disposed of down the drain with excess tap water.

**DATA ANALYSIS**

1. Using the mass of naproxen provided by your TA, calculate the concentration of the naproxen stock solution.
2. With the dilution factor, calculate the concentration of naproxen in the reference solution.
3. Plot the absorption spectrum of naproxen (Absorbance vs. Wavelength). Adjust the data range for both axes of the figure to accentuate the peaks that are in the region of your observation wavelength.
4. Compute the average and standard deviation of the absorbance values at each time for each tablet.
5. Plot the dissolution curve for each of your tablets (Average absorbance vs. Time). Add error bars to the plot using the error at 95% confidence level.
6. Add a new data series into the plot to show the absorbance of the reference solution as a function of time.
7. Use linear regression analysis to evaluate the time dependence of the reference absorbance. Display the results from LINEST analysis. Report the slope, intercept, uncertainties in the slope and intercept, values of *t*_calc_ for the significance of the slope and intercept, *r*^2^, and *F*-value for the significance of the regression equation.
8. If the linear regression is statistically significant, correct the sample dissolution curve with this change. The Correction for Drift is calculated by the fit equation of the reference solution, at each of the dissolution time, and setting the initial value to 1 at time zero.

Calculate the corrected mean absorbance at each time by dividing the experimental mean absorbance of the release solution with the corresponding value in Correction for Drift. Compute the associated standard deviation in each absorbance value.

If the linear regression is statistically insignificant, set all Correction values to 1.

1. Using the molar absorptivity that you calculated in Experiment 4, and the spectrum you collected for the reference solution in this experiment, compute the molar absorptivity of naproxen at the wavelength used in the dissolution experiment (*λ_max_*), and its associated error.
2. Conversion of absorbance values to released naproxen concentrations: Calculate the concentration of naproxen released into the medium based on the molar absorptivity, at each release time, and the standard uncertainty of the concentration.
3. Identify the concentration at infinite time, or the concentration at the plateau of the release curve. Calculate the percent release and its associated standard uncertainty, for each release time.

**NLLS Modeling**

1. Adding Solver to Excel: In the File tab of the Excel file, select Options. In the Excel Options window that appears, click Add-ins. In the list of features, select Solver Add-in and click Go. The solver will be added to Excel by checking the Solver Add-in box in the selection window and clicking OK. Now click on the Data tab, and you will see the Solver for data analysis.

On a Mac, the procedure is slightly different. Open the Excel program for Mac 2016. Under the Tools menu, you will select "Add-ins". Then check "Solver Add-in" and "Analysis ToolPak" and click OK.

1. NLLS Fitting: Follow the detailed instructions in the lecture to perform nonlinear least squares fitting of the dissolution data to the Power Law of drug release, for each of the tablets.
2. Plot the release curve for each tablet (percent release vs. time), using scatter plot with curved lines connecting the data points.
3. Identify the data points corresponding to the first 60% of the dissolution. These are the points that will be used in the NLLS modeling.
4. Overlay the fitting model to the release plot. Make a second figure for the residual plot (deviation vs. time). Keep both figures in the Calculation worksheet for now. Start NLLS fitting and observe how the fit curve approaches the experiment data during the fitting process and how the residual plot changes. Once the NLLS fitting is complete, move the figure into the appropriate sheets designated for these plots.

## hints and other Useful Information

- 1. To accurately measure the time dependence in the dissolution measurements, each cycle of stopping the stirrer-withdrawing solution-replenishing the medium-restarting stirring needs to be done right on the time points (0, 5, 10, 15,… minutes) and performed quickly.
  2. The absorbance of the solutions must be measured at the absorption peak at the longest wavelength. The small molar absorptivity at this wavelength will keep the absorbance values in range when the entire tablet is dissolved and allow the observation of the complete dissolution curve.

## Discussion Questions

1. Using the manufacturer’s specification of 220 mg of naproxen sodium in each tablet, calculate the estimated concentration at 100% release. What is the corresponding absorbance of the solution? Calculate the 95% confidence interval in this estimated absorbance. Is this consistent with the solution absorbance at 100% dissolution of the tablet?
2. In kinetic experiment, it is necessary to determine if a drift occurred during the course of the experiment. What would be the influence to the kinetic model if there is a significant drift in the measured values and the drift is not corrected for?
3. Why is an iterative searching process employed in nonlinear least squares fitting of experimental data to nonlinear models, but is not used in linear regression?
4. What would be the consequence on kinetic analysis if the release medium is not replenished each time after 5 mL of release solution is removed for testing?
5. In Experiment 4, we tested the standard deviations for all calibration solutions. Make a plot of the standard errors in the fractional release over time for this experiment. Describe what you observe. What could be responsible for this phenomenon?
6. Comment on the goodness of fit for the NLLS modeling of your experiment data.
7. Drug release measurements are employed to test if new formulations of drug products are consistent with established standards. Let’s consider release curve for tablet 1 as the standard, and tablet 2 is a new formulation; calculate the similarity and difference factors between the curves. Would you conclude that the two formulations are consistent with each other?

## Lab Report

An Excel template for the lab report is provided on ICON. Turn in the completed spreadsheet to the ICON Assignment for this experiment.

**References**

Harris, D.C, Quantitative Chemical Analysis, 10^th^ edition, W. H. Freeman and Co., New York; 2020.

Ocean Optics, Inc. “USB4000 Fiber Optic Spectrometer Installation and Operation Manual” Document Number 211-00000-000-02-201604.

United States Pharmacopeia Monographs: Naproxen Sodium Tablets.

Yuksel, N.; Arzu E. Kanık, A.E.; Baykara, T. “Comparison of in vitro dissolution profiles by ANOVA-based, model-dependent and -independent methods” *International Journal of Pharmaceutics* **2000**, *209*, 57-67.

Tiong, N.; Elkordy, A.A. "Effects of liquisolid formulations on dissolution of naproxen" *European Journal of Pharmaceutics and Biopharmaceutics* **2009**, *73*, 373-384.

**Supporting Information**

**V. End of Semester Paper: Review of a Research Article**

**Review of a Research Article**

**(100 Points; due on May 3, 2017)**

A central activity in science is the review of the scientific literature, to understand what has been done in the subject of interest and to stay current of the new developments in the field. In this assignment, we will learn this aspect of the chemical measurements. You will read a research article on chemical measurements and write a review of the article.

Search the literature and find your favorite research article. Read the article carefully. Write a review of the research, including two elements:

1. **Summary of the Research Reported in the Article (70 Points)**
   - Goal of the research (10 Points): What is the overarching goal of the research topic? What are the specific aims the authors set out to achieve in this paper? Talk about the existing gap between the goal of the research field and the status in the field; describe how the authors designed the work in this paper to bridge the gap.
   - Methods of measurements (30 Points): Discuss the measurements that the authors used to achieve the research aims. Explain the principles and instrumentation of the methods.
   - Results and conclusions (30 Points): Summarize the main results of the research and conclusions reached.
2. **Your comments on the Research Reported in the Article (30 Points)**
   - Evaluate the significance of the reported research (10 Points)
   - Comment on the methods used in the article (10 Points): Think about how the research was done, and comment on how the experiments were designed to achieve the aims of the article, and how data analysis led the conclusions. (A flow chart might be helpful.)
   - Suggest possible future directions (10 Points): What new directions has this research opened up? What could be the next interesting step(s) to take following this work? What other interesting applications can this research lead to?

***A copy of the article you selected to discuss must be turned in with your review.***

**Requirements:**

- The review has a page limit of **5 pages, double spaced**. The font size should be 12 points.
- The research article selected should either have been published in the past two years (March 2015 – today) or should be considered to be landmark paper in the field if it was published more than two years ago.
- The research article selected could be from one of the following or equivalent journals:

Science (<http://www.sciencemag.org/>)

Nature journals (<http://www.nature.com/>)

Journals published by the American Chemical Society (<http://pubs.acs.org/>)

(1) Formulary, T. N. *The United States Pharmacopeia*; United States Pharmacopeia Convention, Inc., 1979.

(2) Yuksel, N.; Kanık, A. E.; Baykara, T. Comparison of in vitro dissolution profiles by ANOVA-based, model-dependent and-independent methods. *International journal of pharmaceutics* **2000**, *209* (1-2), 57-67.
